# Green light reduces elongation when partially replacing sole blue light independently from cryptochrome 1a

**DOI:** 10.1111/ppl.13538

**Published:** 2021-09-06

**Authors:** Xue Zhang, Mehdi Bisbis, Ep Heuvelink, Weijie Jiang, Leo F. M. Marcelis

**Affiliations:** ^1^ Key Laboratory of Horticultural Crops Genetic Improvement (Ministry of Agriculture), Institute of Vegetables and Flowers Chinese Academy of Agricultural Sciences Beijing China; ^2^ Horticulture and Product Physiology Group Wageningen University Wageningen The Netherlands; ^3^ Leibnitz Institute for Vegetable and Ornamental Production Germany

## Abstract

Although green light is sometimes neglected, it can have several effects on plant growth and development. Green light is probably sensed by cryptochromes (crys), one of the blue light photoreceptor families. The aim of this study is to investigate the possible interaction between green and blue light and the involvement of crys in the green light response of plant photomorphogenesis. We hypothesize that green light effects on morphology only occur when crys are activated by the presence of blue light. Wild‐type Moneymaker (*MM*), *cry1a* mutant (*cry1a*), and two *CRY2* overexpressing transgenic lines (*CRY2‐OX3* and *CRY2‐OX8*) of tomato (*Solanum lycopersicum*) were grown in a climate chamber without or with green light (30 μmol m^−2^ s^−1^) on backgrounds of sole red, sole blue and red/blue mixture, with all treatments having the same photosynthetic photon flux density of 150 μmol m^−2^ s^−1^. Green light showed no significant effects on biomass accumulation, nor on leaf characteristics such as leaf area, specific leaf area, and chlorophyll content. However, in all genotypes, green light significantly decreased stem length on a sole blue background, whereas green light hardly affected stem length on sole red and red/blue mixture background. *MM*, *cry1a*, and *CRY2‐OX3/8* plants all exhibited similar responses of stem elongation to green light, indicating that cry1a, and probably cry2, is not involved in this green light effect. We conclude that partially replacing blue light by green light reduces elongation and that this is independent of cry1a.

## 1 INTRODUCTION

Leaves reflect a relatively large part of green light (G), causing the green appearance of plants. Green light was for a long time thought to be irrelevant for plant functioning. However, this perception is now fading (Smith et al. 2017). Although leaves appear green, the fraction of green light that is reflected is only about 10–15% (Paradiso et al. 2011; Smith 1986), while the major share (about 75–80%) is absorbed, and the rest transmitted. This suggests that there might very well be a role of green light in photomorphogenesis. Green light may play a major role in controlling plant development in orchestration with red light (R) and blue light (B) (Folta & Maruhnich 2007). Wang and Folta (2013) suggested that this role is particularly important at low light conditions, like a canopy with a high planting density. On the other hand, Terashima et al. (2009) reported that at high photosynthetic photon flux density (PPFD), G drives leaf photosynthesis more efficiently than R and B. This is related to the fact that G can penetrate deep into the mesophyll layers at the single‐leaf level (Smith et al. 2017).

There is increasing evidence for the ability of green light to regulate plant photomorphogenesis. Supplementing G to white light (W) or a mixture of R and B (RB) increased hypocotyl and petiole length in Arabidopsis (Folta 2004; Wang et al. 2015; Zhang et al. 2011). Hypocotyls were longer when G:B ratio was higher (Sellaro et al. 2010). Higher G intensity also increased the content of photosynthetic pigments in Arabidopsis seedlings, and biomass and photosynthetic parameters in leaves of lettuce (Efimova et al. 2013; Golovatskaya & Karnachuk 2008; Johkan et al. 2012; Muneer et al. 2014). Lettuce plants grown in a mixture of R, B and G (RBG) had larger specific leaf area (SLA) but lower stomatal conductance compared with RB alone, where the total light intensity of RBG was higher than that of RB (Kim 2005). Plant height and dry weight increased in cucumbers when adding 520 nm G to a mixture of B, R and far‐red light (RBFrG) compared with RBFr alone of similar light intensity, whereas such effects were not found when adding 595 nm G (Brazaitytė et al. 2009). In a recent review on green light, Battle et al. (2020) indicated that short‐wavelength green light (500–530 nm) may lead to different responses compared to long‐wavelength green light (530–600 nm). Growing lettuce plants at different combinations of G with RB showed that growth increased when the fraction of green light was raised from 0 to 24%, but increasing its proportion from 24 to 86% decreased the growth of leaf area and shoot mass (Dougher & Bugbee 2001; Kim et al. 2004).

The nature of the green light receptor remains controversial, although most researchers proposed that green light is sensed by cryptochromes (crys) (Banerjee et al. 2007; Bouly et al. 2007; Sato et al. 2015). In higher plants, three crys have been described to date: *CRY1* and *CRY2*, both localized predominantly in the nucleus and the cytoplasm (Lin & Shalitin 2003), and *CRY3* in the organelles (Kleine et al. 2003). Two *CRY1* (*CRY1a* and *CRY1b*), one *CRY2* and one *CRY3* (*CRY*‐DASH) genes have been isolated in tomato (Facella et al. 2006; Perrotta et al. 2000, 2001). It has been suggested that green light reverses the action of blue light on the activity of crys, making them inactive for blue light (Banerjee et al. 2007; Bouly et al. 2007). This antagonistic blue‐green interaction was supposed to be mediated through the interconversion of flavin redox states of crys. The authors concluded that the fully oxidized chromophore (FAD) absorbs blue light and is then converted to a semi‐reduced chromophore (FADH), which is the biologically active green‐absorbing form. However, there are some inconsistencies with this proposition. Wang et al. (2013) found that G cannot reverse the cry‐mediated B inhibition of early stem elongation but acts additively with B to drive cry‐mediated inhibition. Sato et al. (2015) found that sole G or sole B during the night period inhibited hypocotyl elongation, which seemed to be mediated by cry2. The carotenoid zeaxanthin has been suggested as a photoreceptor for the stomatal blue light response, which could be reversed when adding G to B, indicating that zeaxanthin might absorb G (Frechilla et al. 1999, 2000). Using different photoreceptor mutants of Arabidopsis, Zhang et al. (2011) concluded that the increased leaf inclination and petiole length induced by supplemental G to RB was mediated neither by crys nor by phytochrome A (phyA) and B (Zhang et al. 2011). A yet unknown green light photoreceptor may exist in plants. While the cryptochrome family has been well studied in the model plant Arabidopsis, information about the crys is limited in crop plants, such as tomato, that has an architecture very different from that of Arabidopsis (Fantini et al. 2019; Liu et al. 2018).

The aim of this study is to investigate the interaction between G and B and the involvement of crys in the green light response of plant photomorphogenesis. We hypothesize that the effect of G on stem elongation only occurs when crys are activated by the presence of B. Experiments in climate rooms were conducted where the effects of 525 nm G were studied by replacing 20% background light of sole B, sole R as well as red/blue mixture. To study the involvement of crys, we used a cryptochrome‐deficient genotype and two genotypes overexpressing crys. In contrast to many other studies on G, we kept the PPFD as well as the ratio of other colors the same when G was added.

## 2 MATERIALS AND METHODS

### 2.1 Plant materials and growth conditions

Tomato (*Solanum lycopersicum*) seeds of wild‐type Moneymaker (*MM*) and two *CRY2* overexpressing transgenic lines (*CRY2‐OX3* and *CRY2‐OX8*, previously named line 52.3 and line 52.8 in Giliberto et al. 2005) were kindly provided by Dr. Elio Fantini, ENEA Trisaia Research Center, Italy. Tomato *cry1a* mutant seeds were obtained from Tomato Genetic Resource Center, UC Davis, USA. Seeds were germinated in vermiculite under darkness for 3 days and then transferred to 150 μmol m^−2^ s^−1^ white light‐emitting diode (LED; GreenPower, Phillips). Day/night temperature was maintained at 22/18°C with a photoperiod of 18 h. Relative air humidity was 70%.

Ten days after sowing, plants were transplanted in 11 × 11 × 12 cm black plastic pots filled with ~6 mm expanded clay grid (4–8 mm; Jongkind hydrocorns) and light treatments started. The treatments consisted of sole blue, sole red, red/blue mixture (red/blue ratio = 3/1) with or without green. Total PPFD was kept at 150 μmol m^−2^ s^−1^ at the top of plants in all treatments. When green was added, the red/blue ratio was kept the same as in the treatment without green light (Table 1). Light was provided by narrow band LEDs with peaks at 447 nm (blue; Greenpower, Philips), 667 nm (red; Greenpower, Philips), and 525 nm (green; Lumileds; Figure S1). PPFD, phytochrome photostationary state (PSS; Sager et al. 1986), and the fraction of red (600–700 nm), blue (400–500 nm), and green (500–600 nm) light in all LED treatments were measured by an Apogee Spectroradiometer SS‐110. These measurements were performed at a 45 cm distance from the light source, and the light source was kept at a 40 ~ 50 cm distance from the top of plants by changing the height of the LEDs twice a week during the growing period. The top of plants of different genotypes was kept at the same level by adjusting the height of pots.

**TABLE 1:** Total PPFD (photosynthetic photon flux density) and PPFD of red (R; 600–700 nm), blue (B; 400–500 nm), and green (G; 500–600 nm) for the six spectral treatments as well as the phytochrome photostationary state (PSS)

Spectral treatment	Light intensity (μmol m^−2^ s^−1^)				PSS
	Total	Red (R)	Blue (B)	Green (G)	
R	150	150			0.880
RG	150	120		30	0.884
B	150		150		0.505
BG	150		120	30	0.578
RB	150	112.5	37.5		0.881
RBG	150	90	30	30	0.877

### 2.2 Measurements

Plants were measured 21 days after transplanting. Stem length was measured up to the apex. Total leaf area was measured using a leaf area meter (model LI‐3000; LI‐COR). Roots, stems and leaves were separated and dried in a ventilated oven at 105°C for 24 h to determine the dry weight (DW). From the above, the specific leaf area (m^2^ of leaf area g^−1^ of leaf DW) was determined.

The fourth leaf counted from the top was used for measuring photosynthetic pigments. Photosynthetic pigments of fresh leaves were extracted in 100% *N,N*‐Dimethylformamide (DMF) and then measured using Varian Cary 4000 spectrophotometer. The equations of Wellburn (1994) were used to determine concentrations of chlorophyll *a* (Chl*a*) and *b* (Chl*b*) as well as total carotenoids (Car) in μg ml^−1^ DMF.

$$\mathrm{Chl}a=12\;A_{663.8}-3.11\;A_{646.8}\mathrm{Chl}b=20.78\;A_{646.8}-4.88\;A_{663.8}\mathrm{Car}=\left(1000\;A_{480}-1.12\;\mathrm{Chl}a-34.07\;\mathrm{Chl}b\right)/245$$

### 2.3 Statistical set‐up and analysis

The experiment was conducted five times after each other, representing five blocks. Six light treatments were applied to four genotypes. In each block, measurements were performed on three individual plants for each combination of light treatment and genotype (nine plants for stem length and leaf number); hence the total number of replicate plants per combination of light treatment and genotype were 15 (45 for stem length and leaf number). A split‐plot design was applied to each block, with light treatment as the whole‐plot factor and genotype as the subplot factor. Analysis of variance (ANOVA) was conducted using Genstat 19.0 for Windows. Residuals were tested for normality (Sapiro‐Wilk test at *p* = 0.05). In the case of non‐normal residuals, the original data were log‐transformed, which always resulted in normal residuals. For mean separation, Fisher's unprotected LSD test at *p* = 0.05 was used; “unprotected” because we also applied this test for testing differences among individual combinations of light treatment × genotype when the *F*‐test for interaction was not significant at *p* = 0.05.

## 3 RESULTS

### 3.1 Green light reduced stem length when partially replacing sole blue light

**FIGURE 1:**
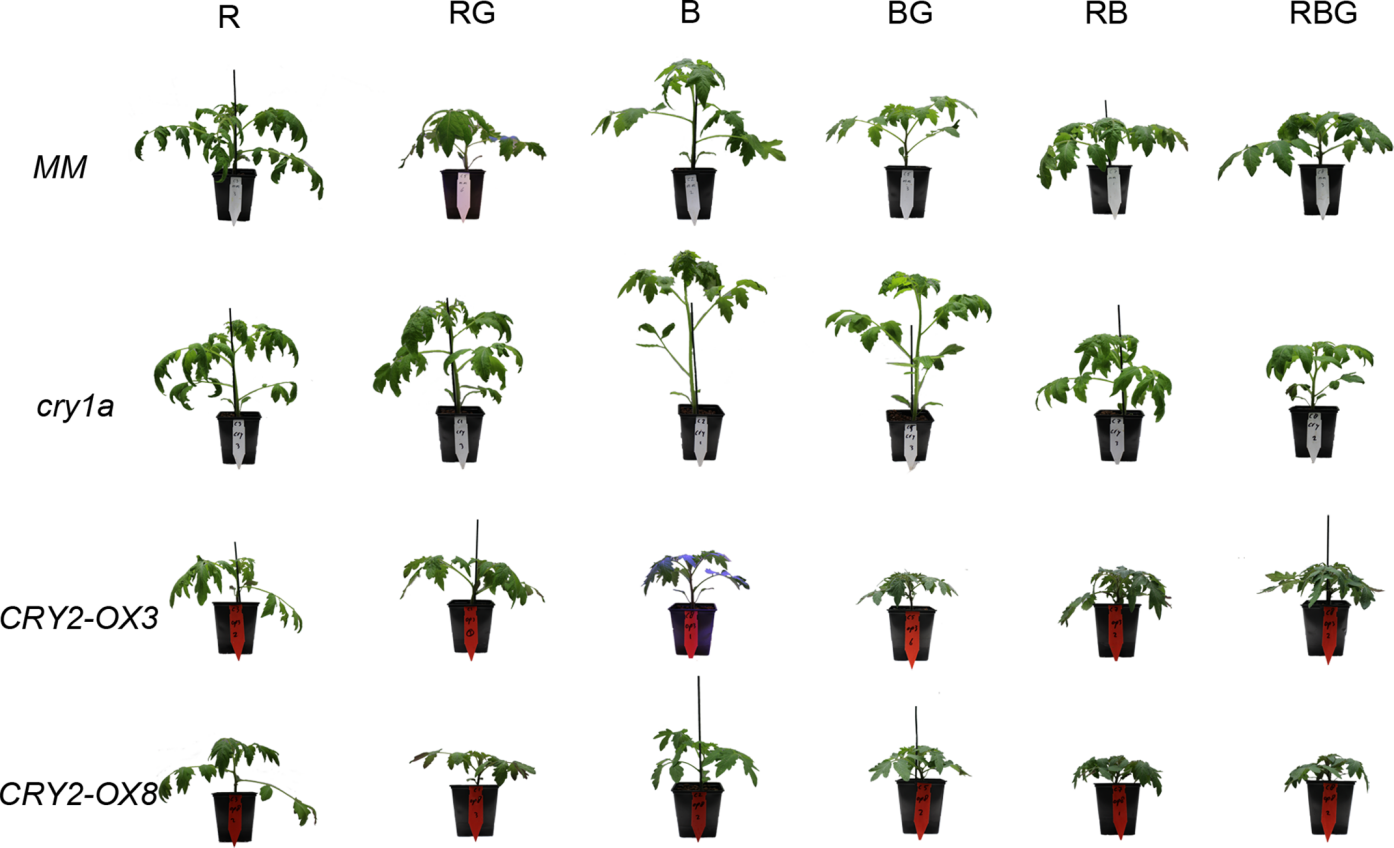
Effect of partially (20%) replacing sole red (R), sole blue (B) or red/blue (RB; ratio 3:1) by green (G) light on the phenotypes of four tomato genotypes: *MM* (Moneymaker, wild‐type), *cry1a* (*CRY1a*‐deficient), *CRY2‐OX3* (*CRY2* overexpressing, line 52.3), and *CRY2‐OX8* (*CRY2* overexpressing, line 52.8). A picture of one representative plant is shown per combination of light treatment and genotype

Figure 1 shows the pictures of representative plants of the four genotypes grown under the six light treatments. Partially (20%) replacing sole B by G (BG) significantly reduced stem length in all four genotypes (Figure 2). Partially replacing RB by G (RBG) did not change stem length in any of the genotypes. Partially replacing sole R by G (RG) did not affect stem length in the wild‐type *MM* and *cry1a* mutant but slightly reduced stem length in the two *CRY2* overexpressors *CRY2‐OX3* and *CRY2‐OX8* (Figure 2). Interestingly, *CRY1a*‐deficient plants were remarkably taller than other genotypes, even under the 100% R background (Figure 2).

**IGURE 2:**
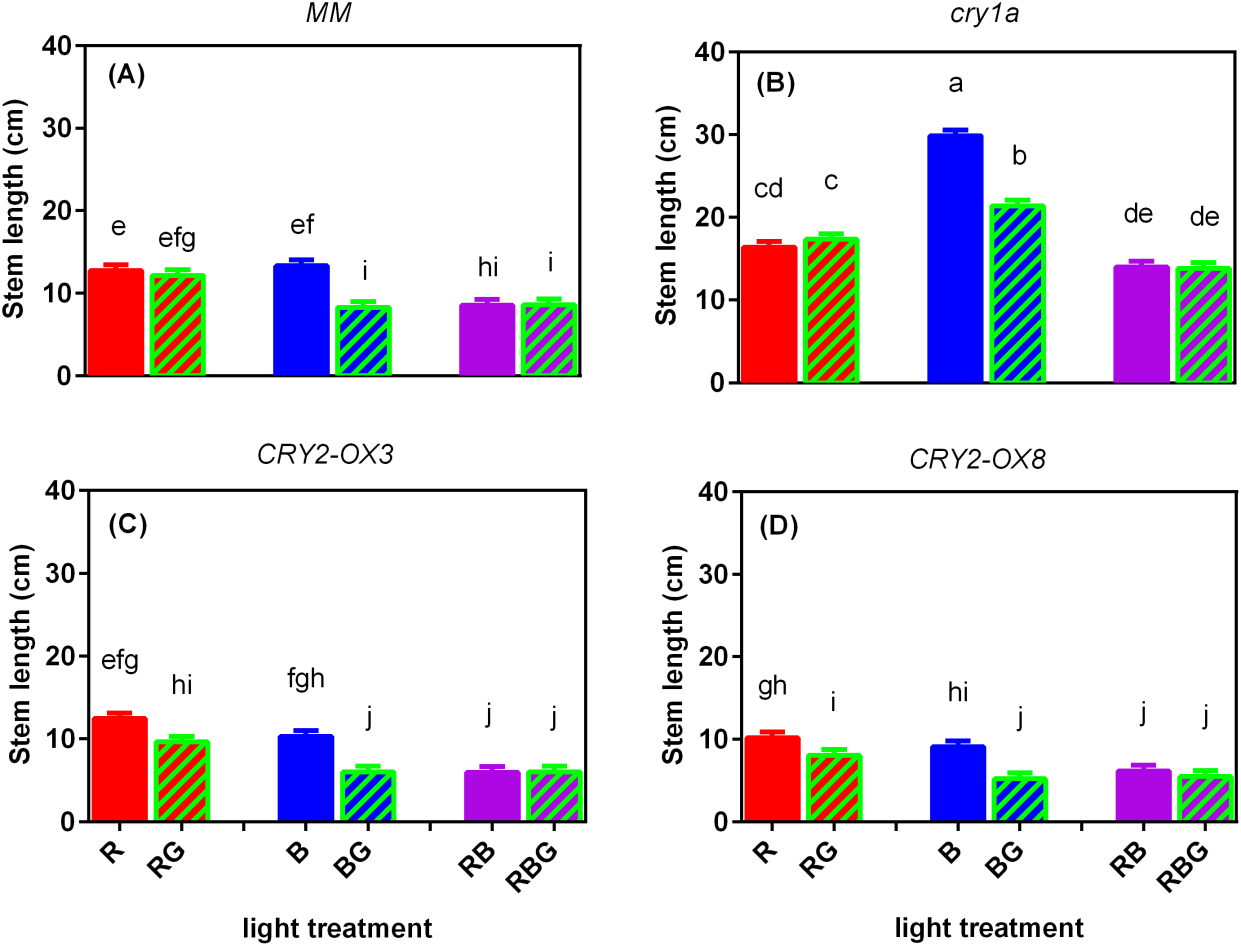
F Effect of partially (20%) replacing sole red (R), sole blue (B) or red/blue (RB; ratio 3:1) by green (G) light on stem length on day 21 after transplanting of four tomato genotypes, (A) *MM* (Moneymaker, wild‐type), (B) *cry1a* (*CRY1a*‐deficient), (C) *CRY2‐OX3* (*CRY2* overexpressing, line 52.3), and (D) *CRY2‐OX8* (*CRY2* overexpressing, line 52.8). There was a significant interaction between light treatment and genotype (log‐transformed data; *p* < 0.001). Different letters above bars indicate significant differences among light treatment × genotype combinations (*p* = 0.05), thus it allows comparison of bars among figures A–D. Vertical bars indicate SE of the mean of five blocks (*n* = 5), each based on nine replicate plants

### 3.2 Green light did not induce changes in specific leaf area but reduced shoot:root ratio of *CRY2* overexpression lines

**FIGURE 3:**
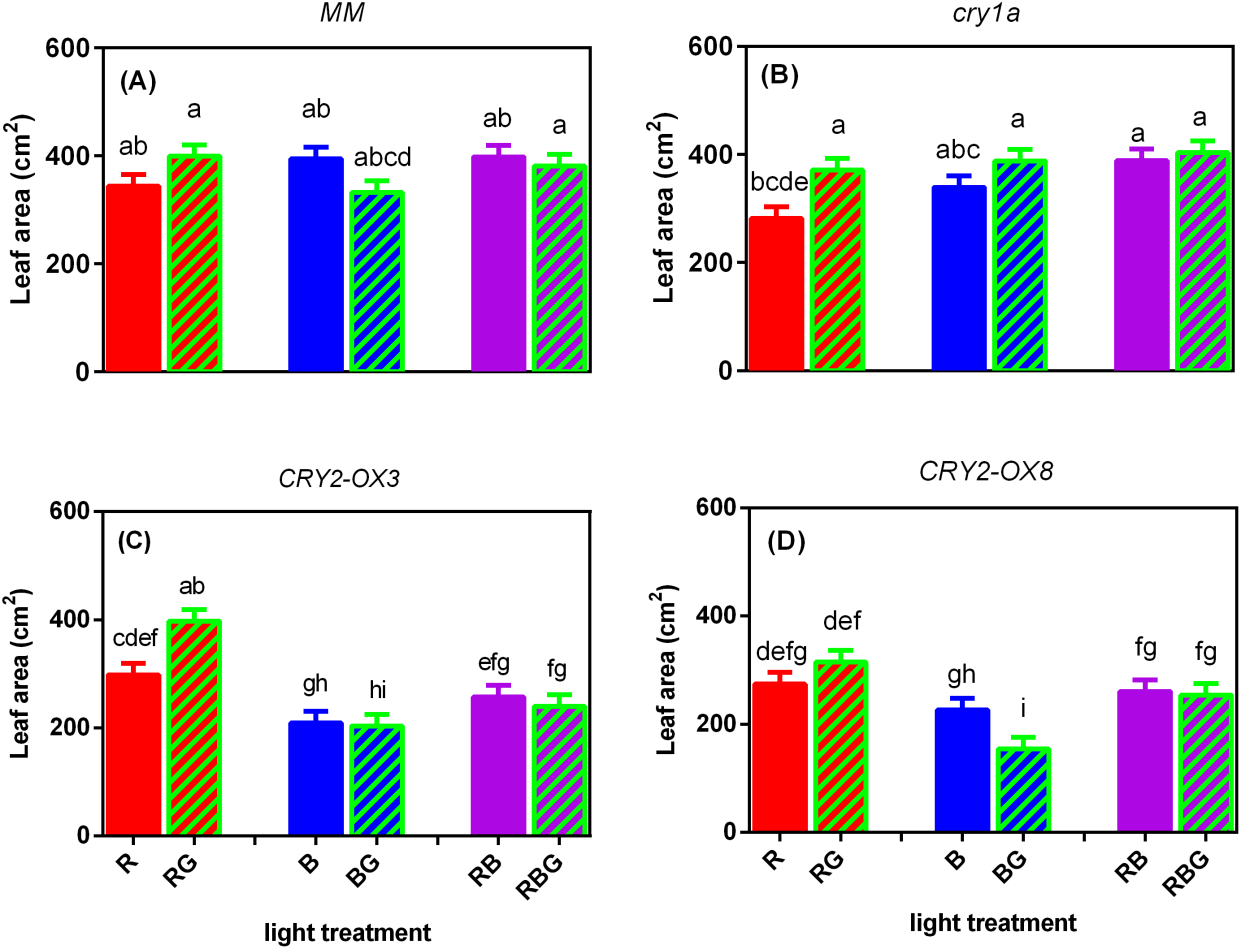
Effect of partially (20%) replacing sole red (R), sole blue (B) or red/blue (RB; ratio 3:1) by green (G) light on leaf area on day 21 after transplanting of four tomato genotypes, (A) *MM* (Moneymaker, wild‐type), (B) *cry1a* (*CRY1a*‐deficient), (C) *CRY2‐OX3* (*CRY2* overexpressing, line 52.3), and (D) *CRY2‐OX8* (*CRY2* overexpressing, line 52.8). Interaction between light treatment and genotype was significant (log‐transformed data; *p* < 0.001). Different letters above bars indicate significant differences between light treatments × genotype combinations (*p* = 0.05), thus it allows comparison of bars among figures A–D. Vertical bars indicate SE of the mean of five blocks (*n* = 5), each based on three replicate plants

No significant interaction between light treatment and genotype was observed in SLA (Figure S3). Partially replacing sole blue and red/blue mixture by green light did not significantly change the leaf area, except that BG remarkably reduced leaf area of *CRY2‐OX8* compared to B (Figure 3). However, partially replacing sole R by G increased leaf area, though this was only significant in *cry1a* mutant and *CRY2‐OX3* (Figure 3). The *CRY2‐OX3* and *CRY2‐OX8* plants tended to have less leaf area than *MM* and *cry1a* mutant in all light treatments, though it was only significant when the background light contained blue light (B, BG, RB, and RBG).

**FIGURE 4:**
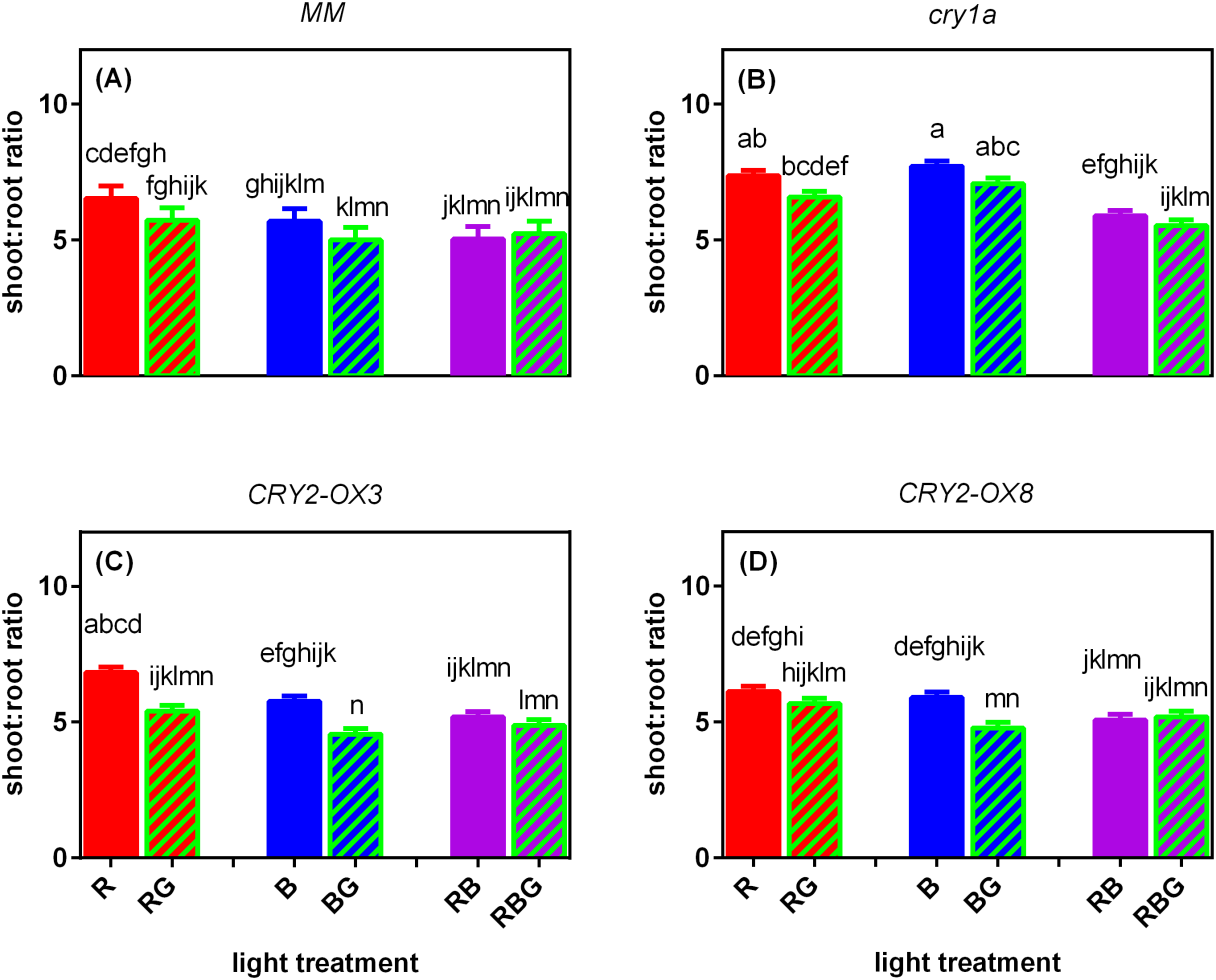
Effect of partially (20%) replacing sole red (R), sole blue (B) or red/blue (RB; ratio 3:1) by green (G) light on shoot: Root ratio on day 21 after transplanting of four tomato genotypes, (A) *MM* (Moneymaker, wild‐type), (B) *cry1a* (*CRY1a*‐deficient), (C) *CRY2‐OX3* (*CRY2* overexpressing, line 52.3), and (D) *CRY2‐OX8* (*CRY2* overexpressing, line 52.8). A significant interaction between light treatment and genotype was observed (*p* = 0.013). Different letters above bars indicate significant differences between light treatment × genotype combinations (*p* = 0.05), thus it allows comparison of bars among figures A–D. vertical bars indicate SE of the mean of five blocks (*n* = 5), each based on three replicate plants

The shoot:root ratio of both wild‐type and *cry1a* mutant did not respond to green light (Figure 4A,B). However, G significantly reduced the shoot: root ratio of *CRY2* overexpressors under sole B background, as well as that of *CRY2‐OX3* under sole R (Figure 4C,D). In line with the results of stem length (Figure 2), *cry1a* mutant had the highest shoot: root ratios, though the effects were only significant under B and BG (Figure 4).

### 3.3 No significant effect of green light on biomass accumulation

**FIGURE 5:**
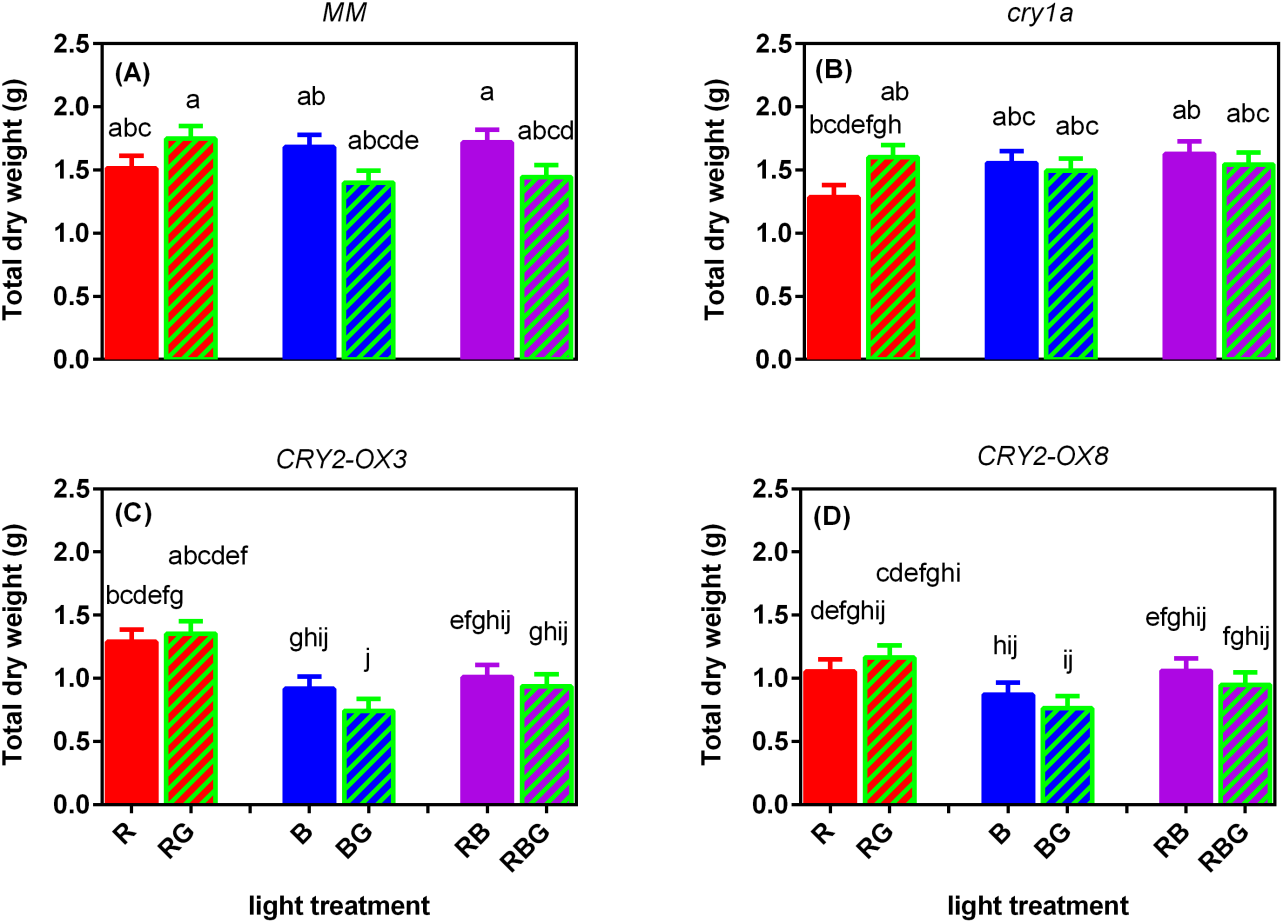
Effect of partially (20%) replacing sole red (R), sole blue (B) or red/blue (RB; ratio 3:1) by green (G) light on total dry weight on day 21 after transplanting of four tomato genotypes, (A) *MM* (Moneymaker, wild‐type), (B) *cry1a* (*CRY1a*‐deficient), (C) *CRY2‐OX3* (*CRY2* overexpressing, line 52.3), and (D) *CRY2‐OX8* (*CRY2* overexpressing, line 52.8). No significant interaction between light treatment and genotype was found (*p* = 0.686), but the effects of light treatment (*p* = 0.04) and genotype (*p* < 0.001) were significant. Different letters above bars indicate significant differences between light treatment × genotype combinations (*p* = 0.05), thus it allows comparison of bars among figures A–D. Vertical bars indicate SE of the mean of five blocks (*n* = 5), each based on three replicate plants

The total dry weight was not significantly affected by partially replacing the different colors (R, B, or RB) by green light, nor was there a significant difference among the genotypes and other spectra (Figure 5). Similarly, the contents of chlorophylls (chls, chl *a* + *b*) and total carotenoids (car), as well as the ratio of chl *a* to chl *b* and chl *a* + *b*/car ratio were mostly not influenced by the genotypes and light treatments (Figure S4). However, partially replacing sole R by G significantly increased the chl *a* + *b*/car ratio of the *cry1a* mutant.

## 4 DISCUSSION

### 4.1 Partially replacing sole blue light by green light reduced elongation independent of cry1a

Tomato stem length was significantly reduced by partially (20%) replacing sole blue light by green light, whereas partially replacing sole red or red/blue mixture with G had little effect on stem length (Figure 2). These effects were due to the elongation of internodes as leaf number was not affected (Figure S2). *CRYs* were reported to mediate hypocotyl elongation inhibition driven by sole blue light or sole green light compared to darkness in Arabidopsis, and G acts additively with B to drive cryptochrome‐mediated inhibition of elongation (Wang et al. 2013). *CRY1a*‐deficient and *CRY2* overexpressing lines (*CRY2‐OX3* and *CRY2‐OX8*) showed similar responses of stem length to partially replacing sole B by G than the wild‐type *MM* (Figure 2). Hence, our results indicated that this green light response was independent of cry1a, probably independent of cry2 as well.

The stem length of the *cry1a* mutant was remarkably longer than the other genotypes under the same light treatment (Figure 2), confirming the involvement of *CRY1a* in the inhibition of internode elongation (Ninu et al. 1999). The overexpression of *CRY2* in *CRY2‐OX3* and *CRY2‐OX8* induced shorter stems (Figure 2), also confirming the involvement of cry2 under all light treatments (Yang et al. 2017).

Through blue light, the neutral FAD chromophore in crys is converted into an active state (FADH) absorbing green light, which converts the crys into a fully reduced and inactive state (Lin & Shalitin 2003; Banerjee et al. 2007; Bouly et al. 2007). Green light partially inhibits cry2 oxidation by blue light (Banerjee et al. 2007; Bouly et al. 2007; Frechilla et al. 2000; Zeugner et al. 2005), contributing to reduced levels of FADH. However, this photocycle model could not explain all interactions between blue and green light on stem length, like the finding that G could also act additively to B to inhibit cry‐mediated stem elongation in Arabidopsis (Wang et al. 2013). In contrast to several other studies on the role of crys, where Arabidopsis seedlings (including cry‐null mutants) received light for very short periods (e.g. 30 min in the works of Banerjee et al. and Bouly et al. 2007), our study was conducted with larger tomato plants that were exposed to different light spectra for a number of weeks. In such long‐term experiments, the responses of the measured parameters (leaf expansion, stem growth, etc.) can be under the control of many photoreceptors and many cellular pathways (Hammad et al. 2020). Therefore, apart from the direct effects of green light on crys, indirect effects can also play a role. In our study G induced a similar response of stem elongation in *CRY2* overexpressing lines than in the wild‐type (Figure 2), confirming that the mechanism underlying crys activation during plant growth has not been elucidated.

Another interpretation of the G‐reduced elongation when partially replacing sole B is that G may activate phytochromes, as also suggested by the increase in PSS value (Table 1). Partially replacing sole B by R also remarkably reduced elongation (Figures 1 and 2), suggesting a potent cry‐phy interaction. Battle et al. (2020) summarized the reported interactions between blue and green light, indicating that green light could act to complement or antagonize blue light‐induced responses dependent on the wavelength of the green light, either through the direct repression of cryptochrome signaling or via a phytochrome‐dependent mechanism.

### 4.2 The involvement of *CRY2* in regulating plant photomorphogenesis

In contrast with *MM* and *cry1a* mutant, stem length was reduced in *CRY2‐OX3* and *CRY2‐OX8* when partly replacing sole R by G (Figure 2), while partly replacing B by G induced a lower shoot: root ratio and smaller leaf area (not significant in *CRY2‐OX3*; Figures 3 and 4). These results indicate the involvement of *CRY2* in green light effects on stem length, shoot: root ratio and leaf area. However, it is not easy to interpret why G did not affect the stem length of *CRY2* overexpressors when partially replacing RB mixture. Maybe this effect was absent because these genotypes had quite short stems when grown under RB compared to sole R or B.

Comparing the tomato *CRY2* overexpressing lines with wild‐type plants, *CRY2* may control vegetative development and photosynthesis as suggested by high‐throughput transcriptomic and proteomic analyses by Lopez et al. (2012), and by the overproduction of chlorophylls in *CRY2* overexpressors (Giliberto et al. 2005). However, we did not observe significant differences in SLA and chlorophyll content between *CRY2‐OX3*/*OX8* and *MM* (Figures S3 and S4). We conclude that the effects of *CRY2* on phenotype are limited, which might result from its redundant role with *CRY1a*.

### 4.3 *PHYs* play a role in blue light effects on elongation

Besides mediation by *CRYs*, the blue light effects might also be mediated by *PHYs*. The PSS value, which is an indicator of phytochrome status, was lower under sole blue than that under all other light treatments; green light had little effect on the PSS value (Table 1). *CRYs* and *PHYs* converge blue and red light signals at different levels to co‐regulate physiological responses, such as root greening, de‐etiolation, shade avoidance symptoms, photoperiodic flowering, etc (Su et al. 2017). Although many studies report that an increasing fraction of blue light reduces stem length (e.g. Kalaitzoglou et al. 2021) due to the involvement of crys, stems under sole B were not the shortest (Figure 2). This may suggest a possible role for phytochrome action. In the *cry1a* mutant, the effects of blue light on elongation via crys are expected to be minor. Hence, the blue effects on stem elongation in this mutant are mainly mediated by phys, resulting in the tallest plants under sole blue light.

Strikingly, similar to 100% B, 100% R also induced significantly longer *cry1a* mutant plants compared to *MM* (Figure 2), consistent with the results of Fantini et al. (2019). On the contrary, Ninu et al. (1999) found that 8 days old *CRY1a* antisense tomato plants did not show an elongated hypocotyl under red light but under blue light (both approximately 8 μmol m^−2^ s^−1^). These differences in results might be caused by the fact that the *CRY1a* gene is not knocked out but only downregulated in *CRY1a* antisense plants, or by differences in the development stage or light intensity. Accumulating evidence in the model plant Arabidopsis has revealed that *CRYs* and *PHYs* share two mechanistically distinct pathways that coordinately regulate transcriptional changes in response to light. However, the role of photoreceptor interactions and the mechanism responsible for the direct convergence of *CRYs* and *PHYs* signals on the *COP1/SPA* complex or phytochrome‐interacting factors (*PIFs*) remain elusive (Su et al. 2017).

In tomato, cryptochrome 1, phytochromes A, B1, and B2 are all capable of mediating responses to B under some circumstances (Weller et al. 2001). In Arabidopsis, *CRYs* may act in a blue‐light independent manner to affect *PHY* regulation of gene expression and development, resulting in different protein expression between the *WT* and *cry1cry2* mutant in red light as well as in blue light (Lopez et al. 2012; Yang et al. 2008). Arabidopsis *CRY1* interacts directly with *PIF4* in a blue light‐dependent manner to repress the transcription activity of *PIF4* (Ma et al. 2016). This indicates that stem elongation in *cry1a* mutants under sole R could be mediated by downstream genes shared by *CRYs* and *PHYs* (Facella et al. 2012; Su et al. 2017). However, the extent and relative importance of their individual contributions differ depending on irradiance, which other photoreceptors are present, and which plant process is examined.

### 4.4 Replacing 20% of red, red/blue, or blue light by green had no significant effect on biomass production

McCree (1972) measured the instantaneous response of leaf photosynthesis to different spectra, finding that the quantum yield of photosynthesis of green photons (525 nm) can be about 25–30% less than that of red photons (675 nm), while the quantum yield of green is comparable to that of blue photons (450 nm). However, this may not be representative of whole plants or plant communities grown at high PPFD under mixed colors of light. Green light could drive carbon fixation deep within leaves (Sun et al. 1998), even more efficiently than R or B (Nishio 2000), because it could penetrate deep into the mesophyll layers (Smith et al. 2017). In our study, where the light contained 0 or 20% green, the plant biomass production rate was not significantly affected by green light (Figure 5). Similarly, the contents of chlorophyll *a* and *b* and carotenoids, as well as their ratios, were hardly affected by green light (Figure S4).

Partially replacing sole R or B or R/B mixture by green light did not cause differences in leaf area, SLA, shoot: root ratio and biomass of *MM* and *cry1a* mutant. This contradicts previous findings on green light responses, but in those studies PPFD also increased when adding G (Kim 2005; Novičkovas et al. 2012; Samuolienė et al. 2012). Zhang et al. (2011) reported that 40% green light induced a shade avoidance response in Arabidopsis seedlings, whereas 10% did not. Too much G (51%) or too little (0%) decreased lettuce growth, while about 24% resulted in the highest growth rate (Kim et al. 2004). However, in our study, 20% G did not induce such effects, which is comparable to the study of Hernández and Kubota (2015), who analyzed the effect of 28% G in cucumber. Kaiser et al. (2019) found that replacing 32% of a red/blue mixture spectrum by green light significantly increased plant biomass and yield. These different observations among studies suggest that G effects might be genotype‐specific and dependent on and/or interact with other environmental conditions.

Although the effects of light spectrum on biomass production were limited in this study, there were profound effects on plant shoot architecture (e.g. stem length). This can be of practical relevance in horticulture to manipulate shoot architecture.

## 5 CONCLUSIONS

Tomato stem elongation was significantly reduced by green light when it partially replaced sole blue light, which may suggest a role for cryptochrome. However, *cry1a* mutant and *CRY2* overexpressing plants showed similar trends on stem length as the wild‐type. This indicates that this response to green light is probably independent of cry1a and cry2. Moreover, *cry1a* mutant plants were significantly taller than other genotypes under all spectra, whereas *CRY2* overexpressing plants had a much shorter stem. We conclude that cry1a, and probably cry2, are not involved in green light effects on elongation under R and B background of our study.

## AUTHOR CONTRIBUTIONS

Xue Zhang, Ep Heuvelink, and Leo F. M. Marcelis conceived and designed the experiment. Xue Zhang and Mehdi Bisbis conducted the experiment. Xue Zhang, Mehdi Bisbis and Ep Heuvelink analyzed the data. Xue Zhang, Mehdi Bisbis and Leo F. M. Marcelis interpreted the data. Xue Zhang wrote the first draft of the manuscript, large parts were edited by Leo F. M. Marcelis and all authors commented on previous versions of the manuscript. All authors read and approved the final manuscript.

## Supporting information


**FIGURE S1** Relative spectral distributions of the red, blue and green narrow band and combined LEDs.
**FIGURE S2** Effect of partially (20%) replacing sole red (R), sole blue (B) or red/blue (RB; ratio 3:1) by green (G) light on leaf number on day 21 after transplanting of four tomato genotypes, (A) *MM* (Moneymaker, wild‐type), (B) *cry1a* (*CRY1a*‐deficient), (C) *CRY2‐OX3* (*CRY2* overexpressing, line 52.3), and (D) *CRY2‐OX8* (*CRY2* overexpressing, line 52.8). No significant (n.s.) effects of green light were found. Vertical bars indicate SE of the mean of three blocks (n = 3), each based on nine replicate plants.
**FIGURE S3** Effect of partially (20%) replacing sole red (R), sole blue (B) or red/blue (RB; ratio 3:1) by green (G) light on specific leaf area on day 21 after transplanting of four tomato genotypes, (A) *MM* (Moneymaker, wild‐type), (B) *cry1a* (*CRY1a*‐deficient), (C) *CRY2‐OX3* (*CRY2* overexpressing, line 52.3) and (D) *CRY2‐OX8* (*CRY2* overexpressing, line 52.8). No significant interaction between light treatment and genotype was found (log‐transformed data; *p* = 0.283) but the effects of light treatment (log‐transformed data; *p* = 0.049) and genotype (log‐transformed data; *p* = 0.002) were significant. Different letters above bars indicate significant differences among light treatment × genotype combinations (*p* = 0.05), thus it allows comparison of bars among Figures A–D. Vertical bars indicate SE of the mean of five blocks (*n* = 5), each based on three replicate plants.
**FIGURE S4** Effect of partially (20%) replacing sole red (R), sole blue (B) or red/blue (RB; ratio 3:1) by green (G) light on chlorophyll content on day 21 after transplanting of four tomato genotypes, *MM* (Moneymaker, wild‐type), *cry1a* (*CRY1a*‐deficient), *CRY2‐OX3* (*CRY2* overexpressing, line 52.3) and *CRY2‐OX8* (*CRY2* overexpressing, line 52.8). (A ~ D) chlorophyll *a* (chl *a*) content; (E ~ H) chlorophyll *b* (chl *b*) content; (I ~ L) carotenoids (car) content; (M ~ P) chl *a* + *b*/car ratio; (Q ~ T) chl *a*/*b* ratio. A significant interaction between light treatment and genotype was found on chl *a*/*b* ratio (*p* = 0.044) and chl *a* + *b*/car ratio (log‐transformed data; *p* = 0.02). No significant interaction between light treatment and genotype was found on chl *a* (*p* = 0.229), chl *b* (log‐transformed data; *p* = 0.234), and car content (*p* = 0.36), but the effects of light treatment (*p* = 0.001; *p* < 0.001; *p* = 0.002) and genotype (*p* < 0.001; *p* < 0.001; *p* < 0.001) were significant. Different letters above bars indicate significant differences among light treatment × genotype combinations (*p* = 0.05), thus it allows comparison of bars among figures A–D, E–H, I–L, M–P, and Q–T, respectively. Vertical bars indicate SE of the mean of five blocks (*n* = 5), each based on three replicate plants.

## Data Availability

The data that support the findings of this study are available from the corresponding author upon reasonable request.
