# Supplementary material for: Green light reduces elongation when partially replacing sole blue light independently from cryptochrome 1a
Source: Physiol Plant. 2021 Sep 6;173(4):1946–55. doi: 10.1111/ppl.13538 (PMC9293030; doi:10.1111/ppl.13538)
Supplement: Supplementary file 1 — FIGURE S1 Relative spectral distributions of the red, blue and green narrow band and combined LEDs. FIGURE S2 Effect of partially (20%) replacing sole red (R), sole blue (B) or red/blue (RB; ratio 3:1) by green (G) light on leaf number on day 21 after transplanting of four tomato genotypes, (A) MM (Moneymaker, wild‐type), (B) cry1a (CRY1a‐deficient), (C) CRY2‐OX3 (CRY2 overexpressing, line 52.3), and (D) CRY2‐OX8 (CRY2 overexpressing, line 52.8). No significant (n.s.) effects of green light were found. Vertical bars indicate SE of the mean of three blocks (n = 3), each based on nine replicate plants. FIGURE S3 Effect of partially (20%) replacing sole red (R), sole blue (B) or red/blue (RB; ratio 3:1) by green (G) light on specific leaf area on day 21 after transplanting of four tomato genotypes, (A) MM (Moneymaker, wild‐type), (B) cry1a (CRY1a‐deficient), (C) CRY2‐OX3 (CRY2 overexpressing, line 52.3) and (D) CRY2‐OX8 (CRY2 overexpressing, line 52.8). No significant interaction between light treatment and genotype was found (log‐transformed data; p = 0.283) but the effects of light treatment (log‐transformed data; p = 0.049) and genotype (log‐transformed data; p = 0.002) were significant. Different letters above bars indicate significant differences among light treatment × genotype combinations (p = 0.05), thus it allows comparison of bars among Figures A–D. Vertical bars indicate SE of the mean of five blocks (n = 5), each based on three replicate plants. FIGURE S4 Effect of partially (20%) replacing sole red (R), sole blue (B) or red/blue (RB; ratio 3:1) by green (G) light on chlorophyll content on day 21 after transplanting of four tomato genotypes, MM (Moneymaker, wild‐type), cry1a (CRY1a‐deficient), CRY2‐OX3 (CRY2 overexpressing, line 52.3) and CRY2‐OX8 (CRY2 overexpressing, line 52.8). (A ~ D) chlorophyll a (chl a) content; (E ~ H) chlorophyll b (chl b) content; (I ~ L) carotenoids (car) content; (M ~ P) chl a + b/car ratio; (Q ~ T) chl a/b ratio. A significant [file PPL-173-1946-s001.docx]

**Supporting Information**





**Fig. S1** Relative spectral distributions of the red, blue and green narrow band and combined LEDs.





**Fig. S2** Effect of partially (20%) replacing sole red (R), sole blue (B) or red/blue (RB; ratio 3:1) by green (G) light on leaf number on day 21 after transplanting of four tomato genotypes, (A) *MM* (Moneymaker, wild-type), (B) *cry1a* (*CRY1a*-deficient), (C) *CRY2-OX3* (*CRY2* overexpressing, line 52.3) and (D) *CRY2-OX8* (*CRY2* overexpressing, line 52.8). No significant (n.s.) effects of green light were found. Vertical bars indicate SE of the mean of 3 blocks (n=3), each based on 9 replicate plants.





**Fig. S3** Effect of partially (20%) replacing sole red (R), sole blue (B) or red/blue (RB; ratio 3:1) by green (G) light on specific leaf area on day 21 after transplanting of four tomato genotypes, (A) *MM* (Moneymaker, wild-type), (B) *cry1a* (*CRY1a*-deficient), (C) *CRY2-OX3* (*CRY2* overexpressing, line 52.3) and (D) *CRY2-OX8* (*CRY2* overexpressing, line 52.8). No significant interaction between light treatment and genotype was found (log-transformed data; *P*=0.283), but the effects of light treatment (log-transformed data; *P*=0.049) and genotype (log-transformed data; *P*=0.002) were significant. Different letters above bars indicate significant differences among light treatment x genotype combinations (*P*=0.05), thus it allows comparison of bars among figures A-D. Vertical bars indicate SE of the mean of 5 blocks (n=5), each based on 3 replicate plants.





**Fig. S4** Effect of partially (20%) replacing sole red (R), sole blue (B) or red/blue (RB; ratio 3:1) by green (G) light on chlorophyll content on day 21 after transplanting of four tomato genotypes, *MM* (Moneymaker, wild-type), *cry1a* (*CRY1a*-deficient), *CRY2-OX3* (*CRY2* overexpressing, line 52.3) and *CRY2-OX8* (*CRY2* overexpressing, line 52.8). (A~D) chlorophyll *a* (chl *a*) content; (E~H) chlorophyll *b* (chl *b*) content; (I~L) carotenoids (car) content; (M~P) chl *a*+*b*/car ratio; (Q~T) chl *a*/*b* ratio. A significant interaction between light treatment and genotype was found on chl *a*/*b* ratio (*P*=0.044) and chl *a*+*b*/car ratio (log-transformed data; *P*=0.02). No significant interaction between light treatment and genotype was found on chl *a* (*P*=0.229), chl *b* (log-transformed data; *P*=0.234), and car content (*P*=0.36), but the effects of light treatment (*P*=0.001; *P*<0.001; *P*=0.002) and genotype (*P*<0.001; *P*<0.001; *P*<0.001) were significant. Different letters above bars indicate significant differences among light treatment x genotype combinations (*P*=0.05), thus it allows comparison of bars among figures A-D, E-H, I-L, M-P and Q-T, respectively. Vertical bars indicate SE of the mean of 5 blocks (n=5), each based on 3 replicate plants.
